# Supplementary material for: Molecular mechanism of antihistamines recognition and regulation of the histamine H1 receptor
Source: Nat Commun. 2024 Jan 2;15:84. doi: 10.1038/s41467-023-44477-4 (PMC10762250; doi:10.1038/s41467-023-44477-4)
Supplement: Supplementary file 1 — Supplementary information [file 41467_2023_44477_MOESM1_ESM.pdf]

Supplementary Information for

Molecular mechanism of antihistamines recognition and regulation of the histamine H_1 receptor

Dandan Wang[1#], Qiong Guo[1#], Zhangsong Wu[2#], Ming Li[1], Binbin He[1], Yang Du[2], Kaiming Zhang[1*], Yuyong Tao[1*]

[#]These authors contributed equally.

1. Department of Laboratory Medicine, The First Affiliated Hospital of USTC, MOE Key Laboratory for Membraneless Organelles and Cellular Dynamics, Hefei National Center for Cross-disciplinary Sciences, Biomedical Sciences and Health Laboratory of Anhui Province, Center for Advanced Interdisciplinary Science and Biomedicine of IHM, Division of Life Sciences and Medicine, University of Science and Technology of China, 230027 Hefei, P.R. China.

2. Kobilka Institute of Innovative Drug Discovery, School of Medicine, The Chinese University of Hong Kong, 518172, Shenzhen, Guangdong, China.

*Correspondence: Yuyong Tao, taoyy@ustc.edu.cn; Kaiming Zhang, kmzhang@ustc.edu.cn.

Supplementary Figures 1-14
Supplementary Tables 1-3

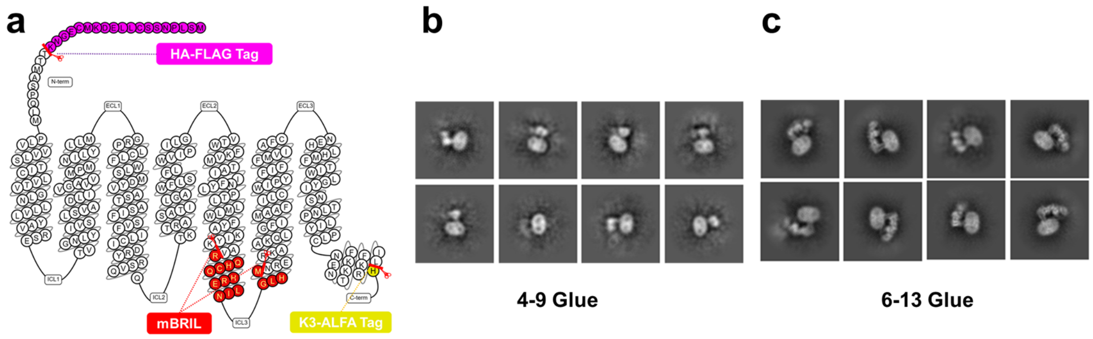

Supplementary Fig. 1. The construct and glue molecule design of the H_1R complex for structural determination.

a. The construct of H_1R for structural determination. Residues in purple, red, and yellow backgrounds were replaced with HA-FLAG tag, mBRIL, and K3-ALFA tag, respectively. **b, c.** Representative 2D classifications of 4-9 glue (**b**) and 6-13 glue samples (**c**). The 6-13 glue shows more desired particles.

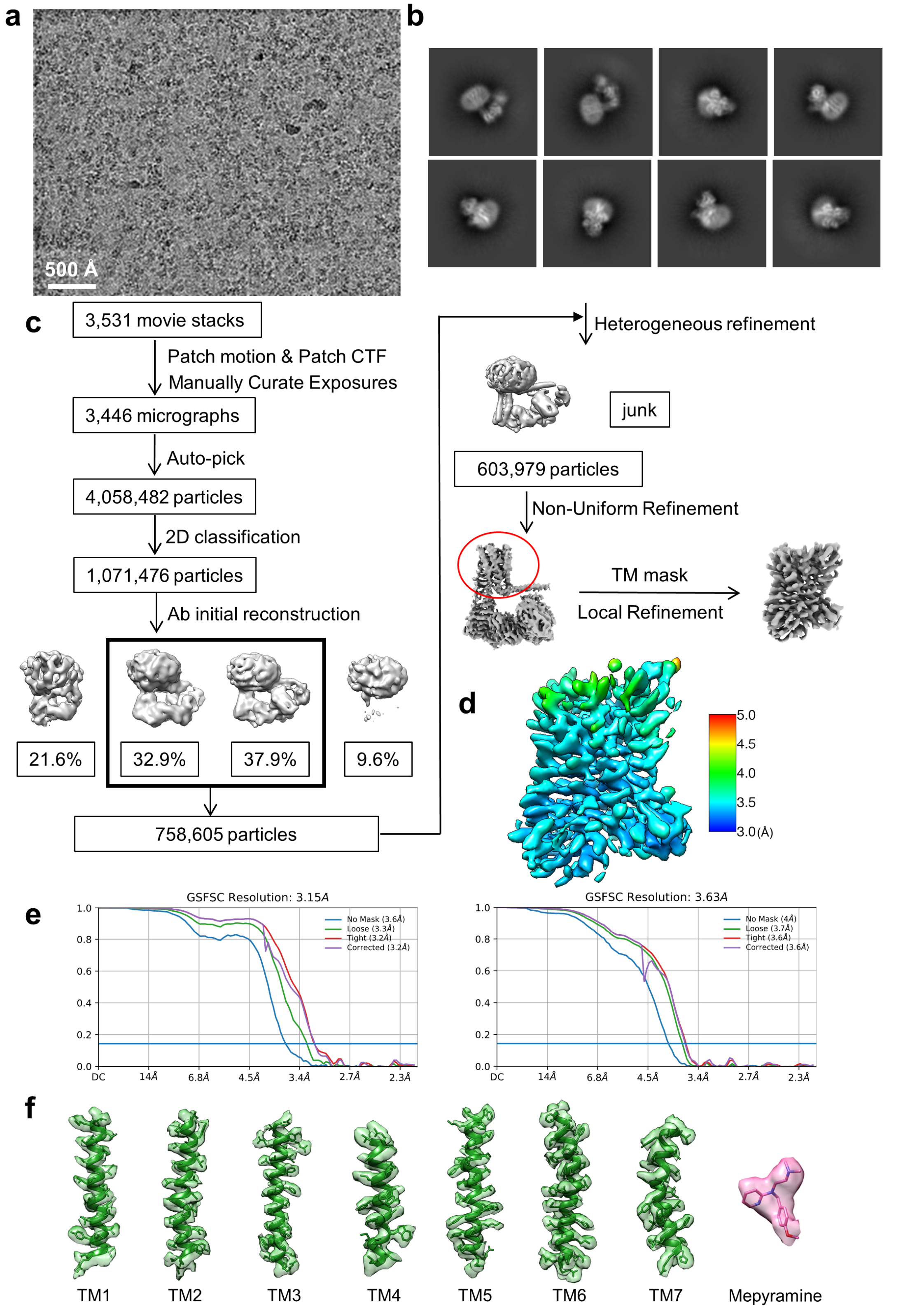

Supplementary Fig. 2. Cryo-EM processing and 3D reconstruction workflow for the H_1R-mepyramine complex.

a. Representative motion-corrected cryo-EM micrograph. **b.** Reference-free 2D class averages. **c.** Workflow of the data processing. **d.** Resolution maps for the final 3D reconstruction of the TM region of H_1R-Mepyramine. **e.** Gold standard FSC plots for the 3D reconstructions of the

whole map (left) and TM region (right), calculated in cryoSPARC. **f.** Cryo-EM density maps and models of the seven transmembrane helices (TM1-7) of H_1R and mepyramine. Maps are shown in green and hot pink, respectively.

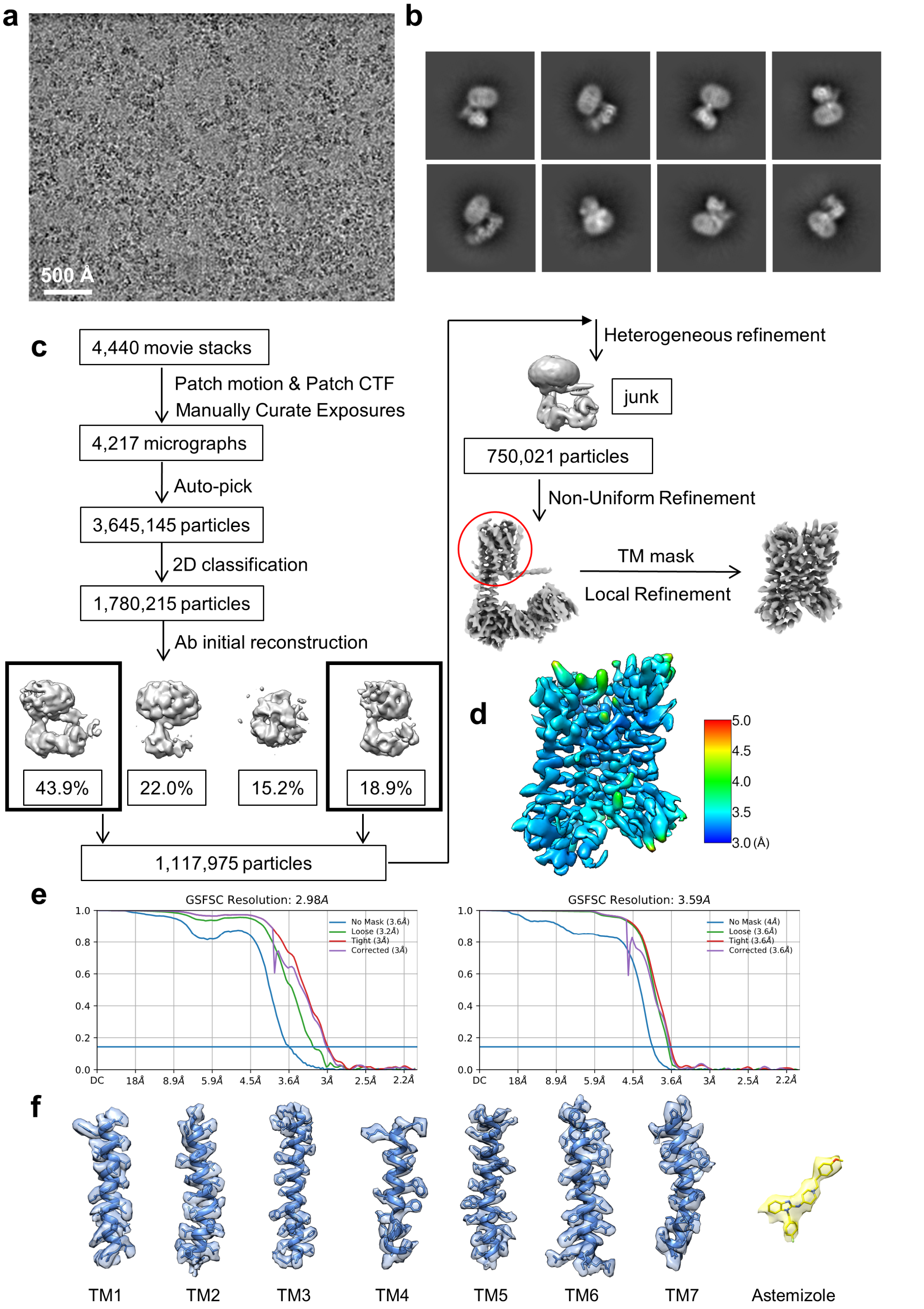

Supplementary Fig. 3. Cryo-EM processing and 3D reconstruction workflow for the H_1R-astemizole complex.

a. Representative motion-corrected cryo-EM micrograph. **b.** Reference-free 2D class averages. **c.** Workflow of the data processing. **d.** Resolution maps for the final 3D reconstruction of the TM region of H_1R-Astemizole. **e.** Gold standard FSC plots for the 3D reconstructions of the

whole map (left) and TM region (right), calculated in cryoSPARC. **f.** Cryo-EM density maps and models of the seven transmembrane helices (TM1-7) of H_1R and astemizole. Maps are shown in sky blue and pale yellow, respectively.

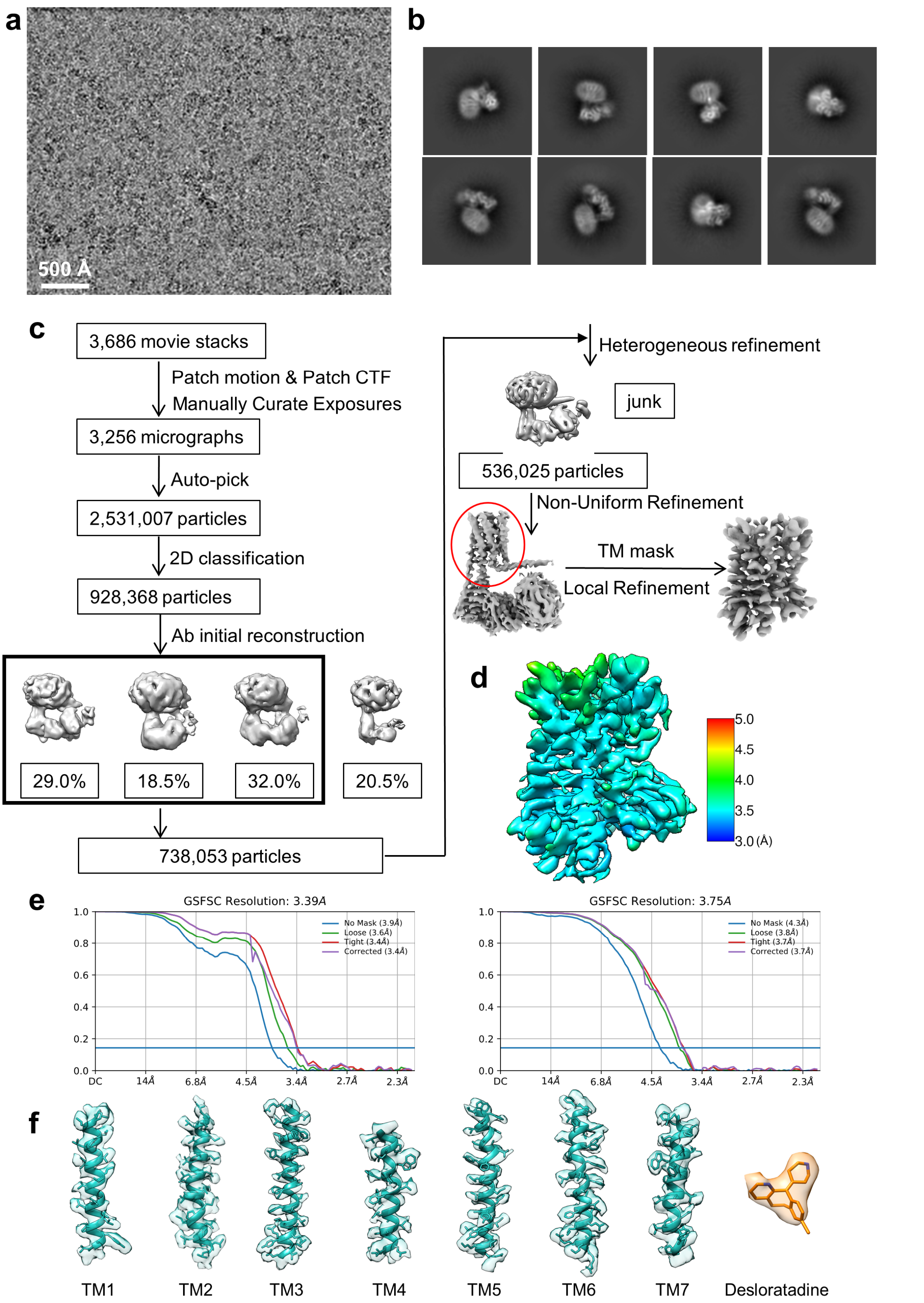

Supplementary Fig. 4. Cryo-EM processing and 3D reconstruction workflow for the H_1R-desloratadine complex.

a. Representative motion-corrected cryo-EM micrograph. **b.** Reference-free 2D class averages. **c.** Workflow of the data processing. **d.** Resolution maps for the final 3D reconstruction of the TM region of H_1R-desloratadine. **e.** Gold standard FSC plots for the 3D reconstructions of the

whole map (left) and TM region (right), calculated in cryoSPARC. **f.** Cryo-EM density maps and models of the seven transmembrane helices (TM1-7) of H_1R and desloratadine. Maps are shown in cyan and orange, respectively.

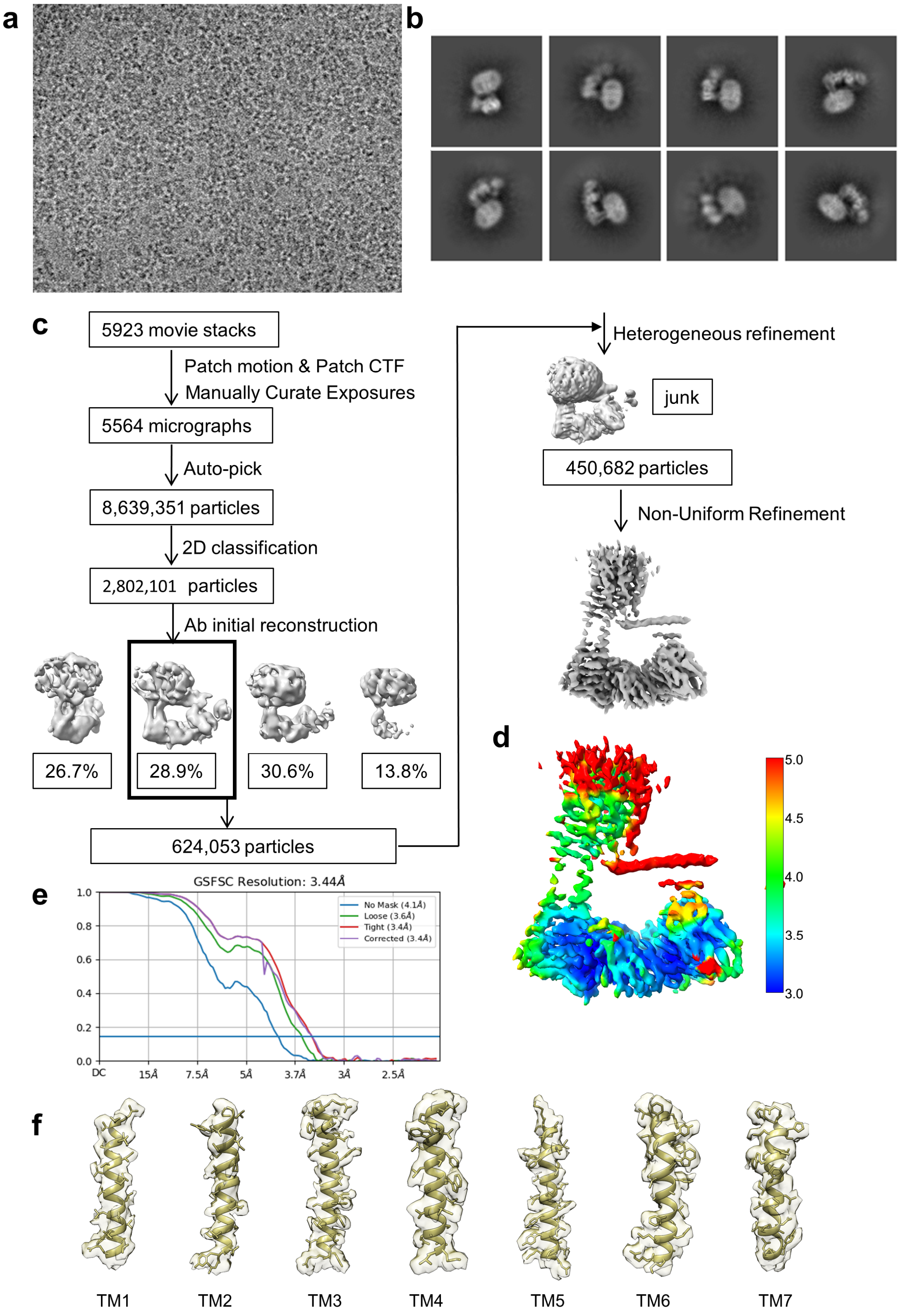

Supplementary Fig. 5. Cryo-EM processing and 3D reconstruction workflow for H_1R in apo form.

a. Representative motion-corrected cryo-EM micrograph. **b.** Reference-free 2D class averages. **c.** Workflow of the data processing. **d.** Resolution maps for the final 3D reconstruction of H_1R in apo form. **e.** Gold standard FSC plots for the 3D reconstructions of the whole map,

calculated in cryoSPARC. **f.** Cryo-EM density maps and models of the seven transmembrane helices (TM1-7) of H_1R in apo form. Maps are shown in olive.

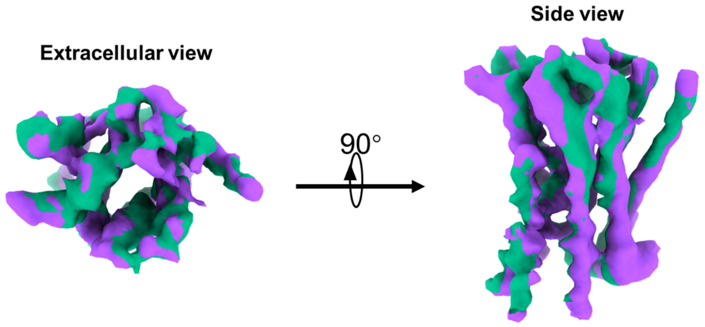

Supplementary Fig. 6. Structural dynamics in apo H_1R.

Two density maps reconstituted from two classes were shown in the 3D variability analysis.

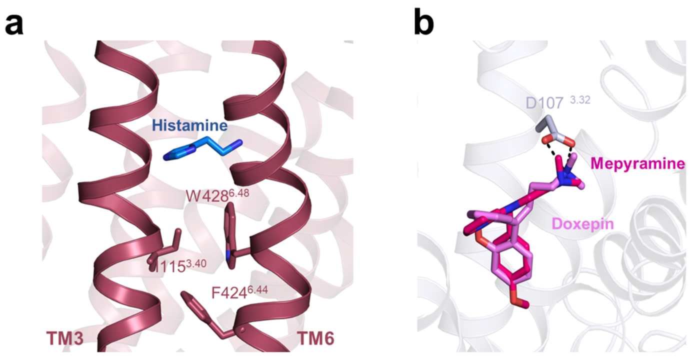

Supplementary Fig. 7. The binding site of histamine and doxepin.

a. The binding site of histamine is above the toggle switch W428$^{6.48}$ and P$^{5.50}$-I$^{3.40}$-F$^{6.44}$ triadmotif (PDB ID: 7DFL). Histamine and H_1R color as marine and raspberry, respectively. **b.** Comparison of the mepyramine-H_1R complex and doxepin-H_1R complex (PDB ID: 3RZE). Doxepin and H_1R are colored violet and gray, respectively. Mepyramine (hot pink) adopts a pose similar to doxepin. Hydrogen bonds are marked as black dashed lines.

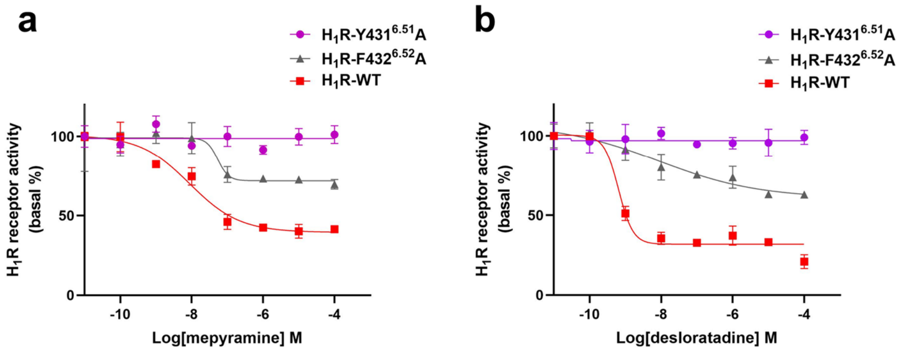

Supplementary Fig. 8. The functional assays in the main binding pocket by mepyramine and desloratadine.

Dose-dependent responses of mepyramine (**a**) and desloratadine(**b**) measured by cellular IP1 accumulation assays in wild-type and mutant H_1R. Data represented as the mean ± SEM, n=3 independent samples.

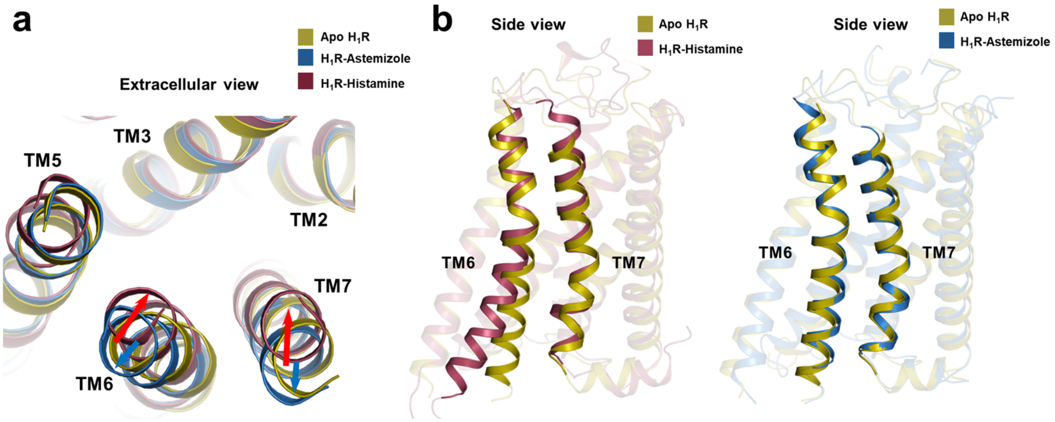

Supplementary Fig. 9. Conformational states of TM6 and TM7 in different structures. Close-up of the TM6 and TM7 structures in apo, histamine-bound (PDB ID: 7DFL) and astemizole-bound structures in top view (**a**) and side view (**b**).

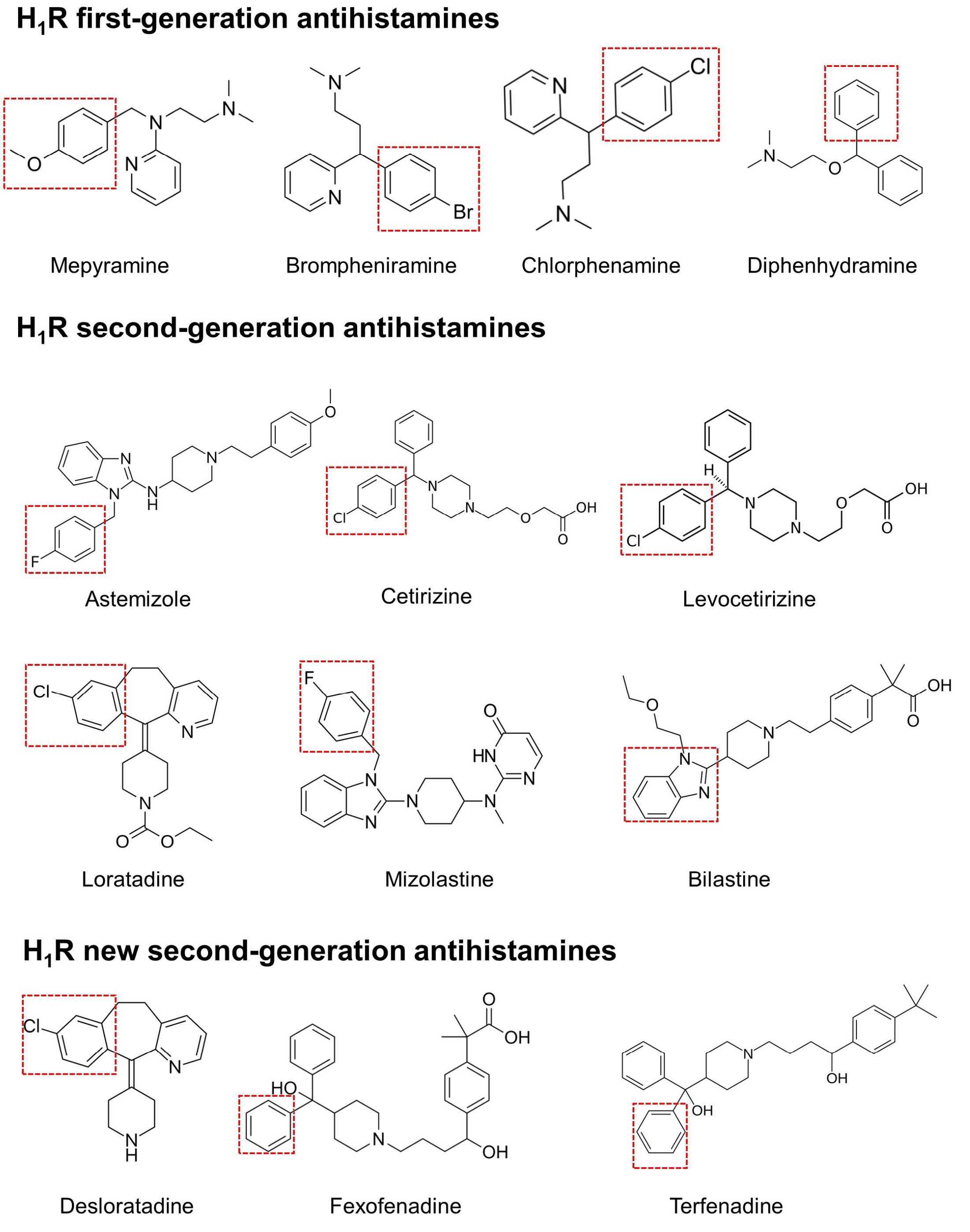

Supplementary Fig. 10. Chemical structures of H_1R antihistamines.

All H_1R antihistamines contain a phenyl group, which is labeled with a red square. Thirteen representative H_1R antihistamines are shown.

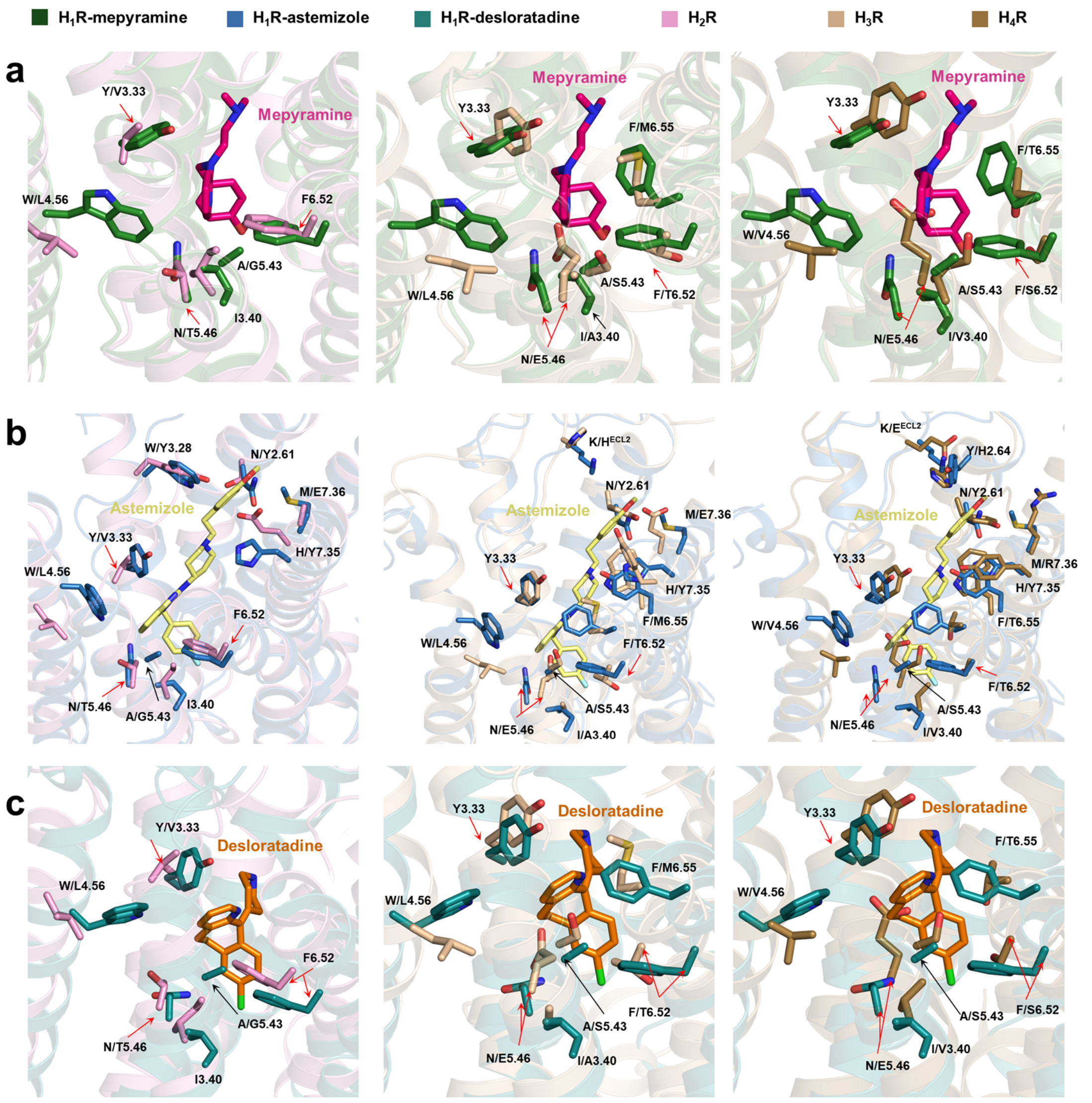

Supplementary Fig. 11. Structural comparison of the ligand pockets in four histamine receptors.

Structural comparison of the H_1R ligand-binding pocket bound to mepyramine (**a**), astemizole (**b**) and desloratadine (**c**) with those from H_2R, H_3R and H_4R. H_1R-mepyramine color as forest, H_1R-astemizole color as sky blue, H_1R-desloratadine color as deep teal, H_2R color as pink (PDB ID: 7UL3), H_3R color as wheat (PDB ID: 7F61) and H_4R color as sand (alphafold model). Varied residues in different subtype receptors are highlighted with arrows.

Ballesteros-Weinstein																			
	Main binding pocket														Secondary binding pocket				
	3.32	3.33	3.37	3.40	4.56	ECL2	5.43	5.46	5.47	6.44	6.48	6.51	6.52	6.55	2.61	2.64	3.28	7.35	7.36
H_1R	D107	Y108	T112	I115	W158	K179	A195	N198	F199	F424	W428	Y431	F432	F435	N84	Y87	W103	H450	M451
H_2R	D	V	T	I	L	K	G	T	F	F	W	Y	F	F	S	Y	Y	E	A
H_3R	D	Y	T	A	L	H	S	E	F	F	W	Y	T	M	Y	Y	W	Y	E
H_4R	D	Y	T	V	V	E	S	E	F	F	W	Y	S	T	Y	H	W	Y	M
$\alpha_{1A}AR$	D	V	T	I	I	I	A	S	F	F	W	F	F	M	S	F	W	F	K
$\alpha_{2A}AR$	D	V	T	I	I	R	C	S	F	F	W	F	F	Y	S	N	Y	F	K
D_1	D	I	T	I	I	N	S	S	F	F	W	F	F	N	K	A	W	F	D
β_1	D	V	T	I	V	C	S	S	F	F	W	F	F	N	G	I	W	F	V
β_2	D	V	T	I	T	C	S	S	F	F	W	F	F	N	G	H	W	Y	I
$5\text{-}HT_{2A}$	D	V	T	I	I	S	S	S	F	F	W	F	F	N	S	T	W	L	N
M_1	D	Y	N	V	L	Q	A	A	F	F	W	Y	N	N	Y	Y	W	W	E

Supplementary Fig. 12. Sequence comparison of the residues from the main and secondary pocket in H_1R-related receptors.

H_1R residues in the main and secondary pockets are compared with equivalent residues from other histamine receptor and aminergenic receptors, the conserved residues are colored with orange background. H_2R: histamine H_2 receptor, H_3R: histamine H_3 receptor, H_4R: histamine H_4 receptor, $\alpha_{1A}AR$: α1A adrenergic receptors, $\alpha_{2A}AR$: α2A adrenergic receptors, D_1: dopamine receptors, $\beta_{1\text{-}2}$: β1-2 adrenergic receptors, $5\text{-}HT_{2A}$: serotonin 5-HT2A receptors, M_1: M1 muscarinic acetylcholine receptors.

H_2R antagonist

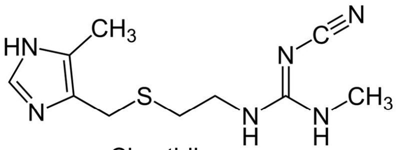

Cimetidine

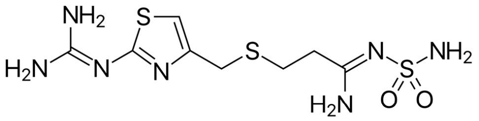

Famotidine

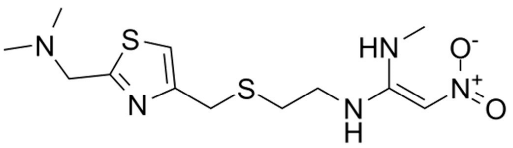

Nizatidine

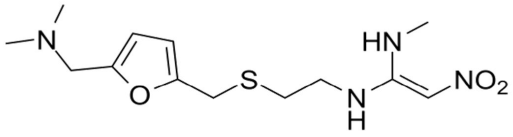

Ranitidine

H_3R antagonist

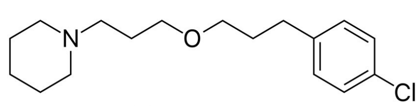

Pitolisant

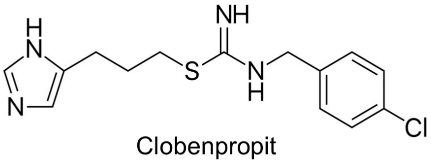

Clobenpropit

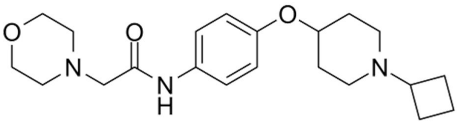

samelisant

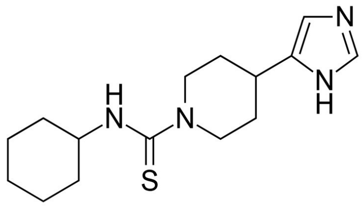

Thioperamide

H_4R antagonist

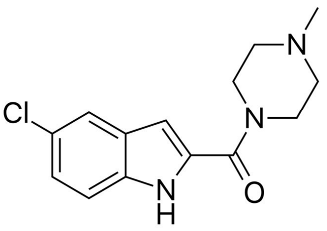

JNJ-7777120

Supplementary Fig. 13. Chemical structures of H_2R, H_3R and H_4R antagonists.
Four representative antagonists of H_2R and H_3R and one antagonist of H_4R are shown.

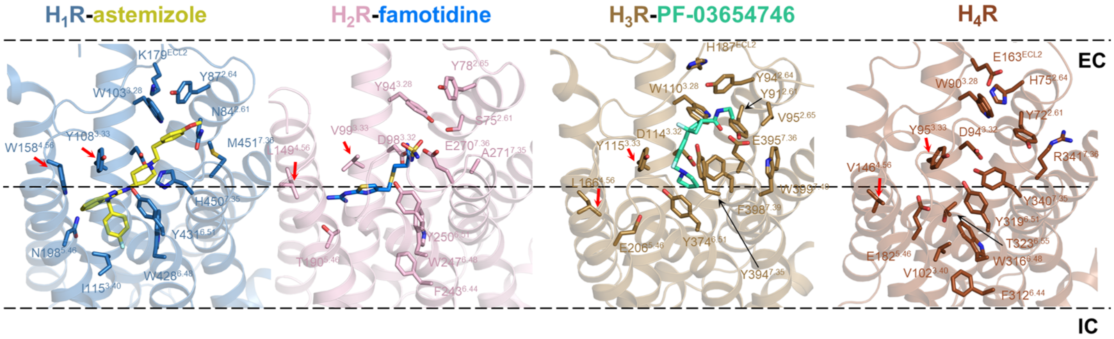

Supplementary Fig. 14. Comparison of the ligand binding positions in H_1R, H_2R, H_3R and H_4R.

Superpositions of the structures of H_1R-astemizole (H_1R in skyblue, astemizole in paleyellow), H_2R–famotidine (PDB: 7UL3, H_2R in lightpink, famotidine in lightblue), H_3R–PF-03654746 (PDB: 7F61, H_3R in sand, PF-03654746 in greenscyan) and H_4R (alphafold model) from the membrane view. The binding positions are clearly different.

Supplementary Table 1. Cryo-EM data collection, model refinement and validation statistics.

**Data collection/ processing**	**apo H_1R-mBRIL**	**Mepyramine-H_1R-mBRIL**	**Astemizole-H_1R-mBRIL**	**Desloratadine-H_1R-mBRIL**
PDB ID	8X5X	8X63	8X5Y	8X64
Magnification	81000	81000	81000	81000
Voltage (kV)	300	300	300	300
Electron exposure (e–/Å^2)	50	50	50	50
Defocus range (μm)	-1.2~-2.0	-1.2~-2.0	-1.2~-2.0	-1.2~-2.0
Pixel size (Å)	0.535	0.535	0.535	0.535
Symmetry imposed	C1	C1	C1	C1
Initial particle projections (no.)	8639351	4058482	3645145	2531007
Final particle projections (no.)	450682	603979	750021	536025
Map resolution (Å)	3.5	3.2	3.0	3.4
FSC threshold	0.143	0.143	0.143	0.143
**Refinement**				
Initial model used (PDB code)	3RZE	3RZE	3RZE	3RZE
Model resolution (Å)	4.1	4.0	3.8	4.2
FSC threshold	0.5	0.5	0.5	0.5
Model resolution range (Å)	50-3.1	50-3.1	50-3.1	50-3.1
Map sharpening B factor (Å^2)	-128.7	-121	-132	-124.6
Model composition				
Non-hydrogen atoms	2107	2254	2294	2191
Protein residues	256	270	274	263
Ligand		1	1	1
B factors (Å^2)				
Protein	98.50	19.07	95.82	71.15
Ligand		14.45	101.80	81.17
R.m.s. deviations				
Bond lengths (Å)	0.003	0.005	0.004	0.005
Bond angles (°)	0.739	0.733	0.775	0.782

Validation				
MolProbity score	1.76	1.98	1.92	2.10
Clash core	10.98	9.42	8.42	13.29
Rotamer outliers (%)	0.00	0.00	0.40	0.00
Ramachandran plot				
Favored (%)	96.75	92.05	92.54	92.55
Allowed (%)	3.25	7.95	7.46	7.45

Supplementary Table 2. Activities of different antihistamines for the wild-type and mutated H_1R in the IP1 assays.

IC50 and Emax estimates represent the average and standard error of mean (SEM) from n=3 independent samples. Emax is defined as percentage of maximum response. N.D. represents no detectable. N.A. represents not available.

H_1R	Mepyramine		Astemizole		Mizolastine		Desloratadine		Loratadine	
	IC50 (nM)	Emax (%)	IC50 (nM)	Emax (%)	IC50 (nM)	Emax (%)	IC50 (nM)	Emax (%)	IC50 (nM)	Emax (%)
**WT**	14.42±8.69	58.56±2.24	16.56±5.06	52.38±6.46	3.84±0.79	51.68±19.4	0.52±0.1	79.06±5	351.5±13.89	64.39±2.53
**Y87A**	N.A.	N.A.	69.97±29.65	40.99±6.4	N.A.	N.A.	N.A.	N.A.	N.A.	N.A.
**W103A**	N.A.	N.A.	165.19±137.21	41.97±12.27	N.A.	N.A.	N.A.	N.A.	N.A.	N.A.
**K179Y**	N.A.	N.A.	31.14±10.63	76.99±0.98	N.A.	N.A.	N.A.	N.A.	N.A.	N.A.
**Y431A**	N.D..	N.D.	N.A.	N.A.	N.A.	N.A.	N.D.	N.D.	N.A.	N.A.
**F432A**	53.91±34.9	30.31±6.93	N.A.	N.A.	N.A.	N.A.	13.1±11.86	37.05±7.27	N.A.	N.A.
**H450A**	N.A.	N.A.	59.29±23.09	71.21±9.27	5.14±0.13	36.1±4.94	N.A.	N.A.	N.A.	N.A.

Supplementary Table 3. The top three docking results of mizolastine and loratadine.

Mizolastine				Loratadine			
Mode	Affinity (kcal/mol)	dist. From best mode		Mode	Affinity (kcal/mol)	dist. From best mode	
		rmsd l. b.	rmsd u. b.			rmsd l. b.	rmsd u. b.
1	-9.0	0	0	1	-7.7	0	0
2	-8.8	3.999	9.499	2	-6.6	4.332	8.862
3	-8.6	1.646	2.012	3	-5.9	4.587	8.552
